# A cross-sectional study using the surveillance, epidemiology, and end results program database to estimate the prevalence of Myelodysplastic Syndrome (MDS) in the United States

**DOI:** 10.1016/j.htct.2026.106507

**Published:** 2026-07-29

**Authors:** Shangyi Fu, Danny Huynh, Milcah Poothakary

**Affiliations:** a Department of Dermatology, Texas Tech University Health Sciences Center School of Medicine, Lubbock, TX, USA; b Full Stack Software Engineer, United Airlines, Houston, TX, USA; c Texas Tech University Health Sciences Center School of Medicine, Lubbock, TX, USA

To the Editor,

Myelodysplastic syndrome (MDS), also referred to as preleukemia, is a group of disorders of bone marrow cells that result in abnormal hematopoiesis. This morphologic dysplasia of hematopoietic cells results in peripheral cytopenias [1]. In this study, we aimed to estimate the prevalence of primary MDS by using the Surveillance, Epidemiology, and End Results (SEER) Program database. SEER is a database supported by the Surveillance Research Program under the National Cancer Institute’s (NCI) Division of Cancer Control and Population Sciences (DCCPS) providing cancer statistics for the U.S. population. The goal of quantifying this disease is to understand its prevalence for future research aimed at reducing the cancer burden in U.S. populations.

At the date of data collection, the SEER database had 43,926,825 participants enrolled (Table 1). We estimated an overall count of 5746.7 affected with MDS. The prevalence was estimated to be highest in the 85 plus age group, showing a pattern that increased with age. The prevalence was also estimated to be highest in White individuals at 0.03%. In Black, American Indian/Alaskan Natives, and Asian or Pacific Islander populations, the prevalence was estimated to be 0.02% (Table 1). It is important to note that the SEER database is composed of 71% White, 12% Black, 2% American-Indian, and 15% Asian-Pacific Islanders [2], which differs slightly from the demographic distribution of the United States, which is 76% White, 14% Black, 1% American-Indian, and 7% Asian [3]. Therefore, our calculation of the prevalence of MDS may underestimate the White and Black populations and overestimate the Hispanic and Asian populations. Moreover, there may be a significant number of unaccounted patients due to factors such as their residency status, limited access to healthcare, and census limitations.

**Table 1:** The prevalence of Myelodysplastic syndrome in the United States, stratified by age and race.

Group	Estimated Prevalence (%)	Estimated Prevalence n	Population at Reference Date	Known Alive	Lost	Lost Estimated Alive	Dead Prior to Reference Date
**Race**							
White	0.03	4455.4	31,240,899	4335	182	120.4	13,873
Black	0.02	499.1	5224,726	484	21	15.1	1125
American Indian/Alaska Native	0.02	47	897,021.5	46	1	1	106
Asian or Pacific Islander	0.02	661.9	6564,178.5	628	60	33.9	1643
Unknown		83.3	0	60	30	23.3	40

**Age at Reference Date (years)**							

00	0.00	1	522,150	1	0	0	0
01–04	0.00	6	2159,900.5	6	0	0	0
05–09	0.00	10.5	2732,193.5	7	4	3.5	3
10–14	0.00	20.6	2786,556.5	17	4	3.6	4
15–19	0.00	30.5	2781,405	28	3	2.5	15
20–24	0.00	25.9	2954,646.5	25	1	0.9	15
25–29	0.00	30.5	3391,241	29	2	1.5	18
30–34	0.00	39.4	3191,280.5	36	4	3.4	15
35–39	0.00	51.8	3049,214.5	46	7	5.8	23
40–44	0.00	57.3	2779,268.5	52	6	5.3	26
45–49	0.00	117.6	2889,978.5	111	7	6.6	65
50–54	0.01	172.1	2837,532.5	161	13	11.1	119
55–59	0.01	272.7	2873,440	258	18	14.7	226
60–64	0.01	422.1	2591,284.5	405	19	17.1	417
65–69	0.03	667.3	2124,581	645	30	22.3	695
70–74	0.04	866	1598,623.5	838	38	28	1114
75–79	0.06	824.1	1077,416	803	34	21.1	1633
80–84	0.09	836	736,037	821	31	15	2177
85+	0.13	1295.3	850,075.5	1264	73	31.3	10,222

A Chi-square test of independence revealed a significant difference in racial distribution between the SEER database and the U.S. Census population (χ² = 3404,209.89; df = 3; N = 304,167,848; p-value <0.001) (Table 2). Given the large sample size, even small proportional differences may reach statistical significance. Although SEER does not perfectly mirror the U.S. population demographically, it remains a widely used, population-based registry for estimating national prevalence. These differences should be considered when interpreting results.

**Table 2:** Comparison of Racial Demographic Distributions between the SEER Program Database and the U.S. Census (2018).

	Estimated SEER Population in 2018	Estimated USA Population in 2018	Row Totals
White	31,240,899 (33,049,304.38) [98,953.07]	197,606,407 (195,798,001.62) [16,702.57]	228,847,306
Black	5224,726 (6661,487.98) [309,883.47]	40,902,223 (39,465,461.02) [52,306.12]	46,126,949
American-Indian	897,022 (478,652.71) [365,678.20]	2417,371 (2835,740.29) [61,723.87]	3314,393
Asian-Pacific Islander	6564,179 (3737,380.93) [2138,071.41]	19,315,021 (22,141,819.07) [360,891.18]	25,879,200
***Column Totals***	43,926,826	260,241,022	**(Grand Total) 304,167,848**

Note: Data are presented as Observed Count (Expected Count) [Cell χ^2^ contribution].

• N (Grand Total): 304,167,848.

• Degrees of Freedom (df): 3.

• Total χ^2^ Statistic: 3404,209.89.

• Significance: p < 0.0001.

Expected counts represent the distribution that would exist if the SEER population perfectly mirrored the U.S. Census proportions. Statistical significance was defined as p-value <0.05.

Altogether, our data suggest that MDS is equally prevalent across various ethnic groups. It also suggests that its prevalence increases with age. With its prevalence in all races, we recommend increased awareness and education regarding MDS in minority patients. It would be beneficial to conduct further epidemiological studies that are not limited by billing codes to validate our findings.

## Disclosures

None.

## Funding

None.

## Conflicts of interest

None.
